# Prevalence of Malnutrition Among Elderly People in Iran: Protocol for a Systematic Review and Meta-Analysis

**DOI:** 10.2196/15334

**Published:** 2019-11-12

**Authors:** Homeira Khoddam, Sepideh Eshkevarlaji, Mahin Nomali, Mahnaz Modanloo, Abbas Ali Keshtkar

**Affiliations:** 1 Nursing Research Center Golestan University of Medical Sciences Gorgan Islamic Republic of Iran; 2 School of Nursing and Midwifery Golestan University of Medical Sciences Gorgan Islamic Republic of Iran; 3 Department of Epidemiology & Biostatistics School of Public Health Tehran University of Medical Sciences Tehran Islamic Republic of Iran; 4 Department of Health Sciences Education Development School of Public Health Tehran University of Medical Sciences Tehran Islamic Republic of Iran

**Keywords:** malnutrition, systematic review, aged, prevalence, Iran

## Abstract

**Background:**

Malnutrition occurs following a decrease or an imbalance in the absorption of energy, protein, vitamins, and minerals because of numerous factors. Thus, it has serious and life-threatening consequences. To plan for this issue, we need information on the burden of this problem.

**Objective:**

The aim of this study is to determine the prevalence of malnutrition among elderly people in Iran.

**Methods:**

For the purpose of this study, papers, including original articles, theses, and conference proceedings on the prevalence of malnutrition among people aged 60 years and above, and have been published in national and international journals until September 2018 will be included without any language limitation. The following keywords along with their synonyms in Persian will be used in the literature search: malnutrition, elderly, and Iran. At first, the screening process will be conducted based on our inclusion and exclusion criteria. Then, the full text of the remaining articles will be read carefully, and eligible articles will be selected according to the objectives of the study. Next, the methodological quality of the selected papers will be reviewed, and the required information will be extracted from those with acceptable quality. Finally, a meta-analysis will be performed using the Stata software (version 14) when optimum criteria are met. It should be noted that all stages of screening, selection, quality assessment of primary studies, and data extraction will be performed by two reviewers independently.

**Results:**

This review is ongoing and will be completed at the end of 2019.

**Conclusions:**

This review aims to provide comprehensive evidence about the prevalence of malnutrition among elderly people in Iran. This can help Iranian health managers and policy makers make informed decisions for preventing malnutrition and promoting the health status of elderly people.

**Trial Registration:**

PROSPERO CRD42018115358; https://tinyurl.com/y28su47m

**International Registered Report Identifier (IRRID):**

DERR1-10.2196/15334

## Introduction

### Description of the Condition

Rapid population growth is a result of positive social, economic, and health trends, which has not only changed the demographic pattern but has also significantly increased the life expectancy from about 48 years in the early 1950s to about 68 years in the early 21st century [1]. Therefore, both developed and developing countries have been faced with a growth in the aging population, which is considered as an important health challenge [2].

In most countries, the chronological age of 60 years has been considered as the beginning of old age [3]. According to the World Health Organization (WHO), the number of older people will reach 1.5 billion by 2050, and the majority of these people will be living in developing countries [1,4]. At the same time, the percentage of people aged over 60 years in Iran is expected to increase from 5.2% (in 2000) to 21.7% (in 2050) [5]. As one of the most important phases of life, old age is accompanied with a wide range of long-term conditions such as chronic illnesses, cognitive problems, physical weakness, anorexia, and chewing and swallowing problems that can disturb the nutritional balance and result in malnutrition [6-11]. Although any age group may suffer from malnutrition, it is most common among older people because of the comorbidities and changes in physiological and psychosocial characteristics of people in this age group [12].

Malnutrition has serious and life-threatening consequences that are known to be major causes of increased mortality among the elderly [13,14]. According to the WHO, nutritional disorders are one of the most common causes of death in the elderly [15]. In addition, complications of malnutrition, including osteoarthritis, osteoporosis, diabetes, cardiovascular disease, and hypertension, impose a significant social and economic burden on elderly people [10,16,17].

The prevalence of malnutrition in elderly people in different countries of the world varies between 10% and 60% [18-33]. Some of these differences are because of the variations in using the measurement tools, study setting, and demographic groups that have been studied [34-36].

Despite the high prevalence reported, it seems that the real prevalence is beyond the reports, as a significant proportion of malnourished individuals are not identified until nutritional deficiencies lead to significant physical changes [34]. On the basis of the issues mentioned above, it seems that the risk of malnutrition among elderly people is a major challenge to the health care systems around the world and needs a special and urgent attention [37].

### Importance of This Review

Over the past decades, despite the scientific community’s desire to conduct studies on the malnutrition of the elderly, there is a large knowledge gap in this area [38]. More importantly, research findings and health practices and policies on this issue are not interconnected, and therefore, informed strategies are not used [37]. In fact, all countries need to integrate the health care policies and researches on the prevention and treatment of malnutrition [35,37].

Iran, like many other countries, has been faced with a remarkable shift in population composition and needs to prepare to address this upcoming challenge. Although some actions such as revisiting the educational system and setting up new care settings for the elderly have been taking place, the role of research findings to inform decisions and policies is neglected.

Several studies have been conducted to determine the prevalence and risk factors of malnutrition in the elderly in Iran [39-50], and these studies have reported different and incomparable prevalence data. The wide discrepancy in the study results limits the use of such evidence for relevant national planning and policy making [29]. We need to synthesize and summarize the evidence on this issue, which would help us bridge the evidence-practice gap and promote the health care decisions and services to elderly people [35]. This aim can be achieved through systematic reviews and meta-analysis of context-based studies.

There are 3 published systematic reviews on malnutrition among elderly people; however, in one of them, hospitalized patients were the studied population [51], and another review had some significant shortcomings such as nonsystematic approach and incomprehensive search [52]. The third one is a systematic review and meta-analysis conducted in 2016 on the malnutrition outbreak among older people in Iran living with families or in nursing homes [26]. This study also had some limitations, including the lack of comprehensiveness in searching and a priori registration or publication of review protocol, using the reporting tool (Strengthening the Reporting of Observational Studies in Epidemiology) to evaluate the risk of bias, and failure to determine the source of heterogeneity. Thus, we designed this study to address the limitations of previous systematic reviews and provide a comprehensive evidence on the prevalence of malnutrition among aged people in Iran.

### Objectives

#### Primary Objective

The primary objective is to assess the prevalence of malnutrition among aged people in Iran.

#### Secondary Objectives

The secondary objectives are (1) to evaluate the prevalence of malnutrition among elderly people in Iran according to the assessment tools, study setting, severity of the problem, and regional setting and (2) to evaluate the heterogeneity and determine its potential causes.

## Methods

This protocol has been reported based on the Preferred Reporting Items for Systematic Reviews and Meta-Analyses Protocol checklist [53].

### Eligibility Criteria

#### Types of Studies

In this systematic review, we will include all studies that reported the prevalence of malnutrition among elderly people in Iran or provided main data to calculate this measure. These may be included as descriptive and observational studies. We will include population-based prevention clinical trials, cohort studies, and case-control studies if the baseline data were available and consistent with the objectives of this systematic review.

#### Types of Participants

We will include studies conducted on people of both genders, aged 60 years and above, who live in elderly nursing homes or are community indwelling elders. Studies on hospitalized elderly people will be excluded. There will be no restriction in terms of race and ethnicity.

#### Type of Measuring Tools

We will include studies that measured and reported malnutrition by using standard and valid measuring tools (questionnaire, anthropometric, or biological indices).

#### Sample Size

We will include studies with a minimum sample size of 30.

### Search Methods for the Identification of Studies

#### Electronic Searches

A comprehensive search will be conducted in local and international databases to obtain published and unpublished articles until September 30, 2018.

PubMed, Scopus, and Web of Science are the international databases that will be searched. In addition, the local databases that will be searched include Iranian Databases of Medical literature, Scientific Information Database, Magiran, Iran Medical Sciences Thesis Database and the Iranian Research Institute for Information Science and Technology.

Furthermore, relevant gray literature (eg, thesis or dissertations and conference papers) will be included by searching in the international and local databases and Google Scholar search engine. We will not consider any language limitation.

We will test the primary search strategy (Multimedia Appendix 1) in PubMed, the main database in the field of medical sciences. At first, we will find equivalent keywords from Medical Subject Headings database and will create appropriate syntaxes for PubMed. Then, we will adopt it for other databases.

#### Searching Other Resources

To ensure access to all relevant articles and to find those not previously found, the list of included articles, references, and the relevant national and international organizations’ reports, such as WHO, will be searched manually.

### Data Collection and Analysis

#### Selection of Studies

The study selection will be done in 2 stages. At first, the search results will be imported into the reference manager software (EndNote X7, Thomson Reuters) for storage and organization. After detecting and deleting duplicates, 2 researchers (SE and HKH) will screen the titles and abstracts of all retrieved documents based on the inclusion and exclusion criteria. Then, the full text of potentially eligible studies will be screened independently by the same researchers to finalize the review and start data extraction. Any disagreement between reviewers will be resolved by discussing or consulting with a third person.

Irrelevant articles, which do not meet the inclusion criteria, will be deleted by providing reasons. All the mentioned steps will be presented on the Preferred Reporting Items for Systematic Reviews and Meta-Analyses (PRISMA) flow diagram [54].

If there is a lack of access to the full text of some studies or if there is any unclear information, the researchers will contact the corresponding author thrice during a period of 7 to 14 days [55,56]. We will report all the missing data if we cannot find them. At the end, all other eligible articles retrieved from other sources will be added to the final list.

#### Data Extraction and Management

At this stage, the 2 researchers will independently extract data from the included articles using data extraction form. The data extraction form includes review information; bibliographic data such as authors, title, year, issue; and study data such as design, setting/context, year, sample size, sampling method, data gathering method, data collection period, participants, and main results. Data extraction will be done by 2 independent reviewers (MM and HKH), and any disagreement will be resolved by discussing or consulting with a third expert person.

#### Assessment of Risk of Bias in Included Studies

The quality assessment and risk of bias of the included studies will be assessed by 2 independent assessors (MN and SE), and the strategy of discussion or consulting with a third reviewer will be applied in case of disagreement. For assessing the observational studies, assessors will use a 10-item rating tool developed by Hoy et al [57] in 2012. This tool has been developed based on literature review, expert opinion, and the researchers’ experiences and has a high interrater agreement in assessing the quality of prevalence studies. On the basis of this, each item scores 0 for the response *No* and 1 for *Yes*. The total score will be between 0 and 10 [57]. We will use the Cochrane Collaboration tool for assessing the clinical trials [58]. The result of risk of bias and quality assessment will be presented in a table.

#### Dealing With Missing Data

We will deal with missing data using 2 strategies. In case of missing the full text of studies from the review, or if there is any unclear information, the researchers will contact the corresponding author(s) thrice during a period of 7 to 14 days [55,56]. When outcome data are not reported, we will calculate the outcome data if there is additional information in the publications [59,60]. However, if these 2 strategies are not successful, we will analyze only the available data and report all missing data and discuss their impact on the findings [59].

#### Assessment of Heterogeneity

We will assess the heterogeneity among studies by using forest plot, I-square (I^2^) test, and Cochran Q test [61-63]. The significance level of less than 0.05 and I^2^ equal to or more than 50% will be assumed as a severe heterogeneity. In cases of severe heterogeneity, subgroup analysis considering age, gender, elderly residency, and malnutrition severity will be performed to identify the potential source of heterogeneity.

#### Assessment of Reporting Bias

We will check the possibility of publication bias graphically (funnel plot) and statistically (Egger test) [61,64], using Stata software, version 12 (StataCorp LP). A *P* value of less than .05 will be considered as statistically significant or nonignorable publication bias.

#### Data Synthesis and Analysis of Outcomes

The main outcome of this review is malnutrition. If there are enough studies reporting the prevalence of malnutrition in elderly people, we will do a meta-analysis to calculate the pooled estimate of prevalence and subgroups analysis (gender, age, and residence place) 95% CIs using Stata software.

On the basis of the level of methodological heterogeneity, we will use fixed or random effects model to conduct meta-analyses [61].

#### Subgroup Analysis and Investigation of Heterogeneity

On the basis of the quality of heterogeneity, subgroup analysis will be conducted on the following variables: age, sex, geographical region, residency (nursing home/home), risk (at risk or affected), and severity of malnutrition. By this way, the potential sources of heterogeneity will be identified.

#### Sensitivity Analysis

Although the need for doing sensitivity analysis is not often specified before completing the systematic review and meta-analysis [61], we will use it to determine the impact of the quality of the study, study design, sample size, and methods of analysis, if needed. In fact, this is a repeated meta-analysis to assess the robustness of the process of selection and inclusion of the studies [65].

## Results

This protocol was registered in the International Prospective Register of Systematic Reviews (PROSPERO) on December 23, 2018. We will report this systematic review and meta-analysis based on the PRISMA checklist. The flow diagram will be drawn to show the systematic search and selection process of articles. In addition, the table of excluded studies and the reasons for their exclusion will be presented.

## Discussion

Systematic reviews and meta-analyses can synthesize and summarize the best knowledge available from health care studies for reducing gaps between evidence and practice [66]. This study presents a protocol for systematic review and meta-analysis targeted to provide comprehensive evidence about the prevalence of malnutrition among elderly people in Iran. The result of this review can provide a context-based evidence to help Iranian health managers and policy makers to make informed decisions for preventing malnutrition and promoting the health status of elderly people.

## Appendix

Multimedia Appendix 1 Search strategy developed for PubMed.

Multimedia Appendix 2 Peer-reviewer report from the Nursing Research Center, Golestan University of Medical Sciences.

## Abbreviations

PRISMA: Preferred Reporting Items for Systematic Reviews and Meta-Analyses
PROSPERO: International Prospective Register of Systematic Reviews
WHO: World Health Organization
