# Supplementary material for: Prevalence of Malnutrition Among Elderly People in Iran: Protocol for a Systematic Review and Meta-Analysis
Source: JMIR Res Protoc. 2019 Nov 12;8(11):e15334. doi: 10.2196/15334 (PMC6880236; doi:10.2196/15334)
Supplement: Multimedia Appendix 1 [file resprot_v8i11e15334_app1.docx]

**Search strategy developed for PubMed**

Malnutrition[All] OR “Nutritional Deficiency”[All] OR “Nutritional Deficiencies”[All] OR Under-nourished[All] OR Undernourished[All] OR Undernutrition[All] OR Malnourish*[All] OR (Status AND Nutritional)[All] OR “Nutrition Status”[All] OR (Status AND Nutrition)[All]) AND (Elderly[All] OR Aged[All] OR Aging[All] OR “old Adult”[All] OR “Elderly people”[All] OR “Old people”[All] OR Senior*[All] OR geriatric[All] OR “Old persons”[All]) AND (Iran[TIAB] OR Iran[AD])
