# Supplementary material for: Prevalence of Malnutrition Among Elderly People in Iran: Protocol for a Systematic Review and Meta-Analysis
Source: JMIR Res Protoc. 2019 Nov 12;8(11):e15334. doi: 10.2196/15334 (PMC6880236; doi:10.2196/15334)
Supplement: Multimedia Appendix 2 [file resprot_v8i11e15334_app2.pdf]

معاونت محترم تحصیلات تکمیلی دانشکده پرستاری و مامایی

با سلام

احتراماً، بدینوسیله پاسخ به موارد خواسته شده توسط داوران محترم پروپوزال پایان نامه ای تحت عنوان: شیوع سوء تغذیه در سالمندان ایران: یک مرور سیستماتیک" خدمتتان ارائه می شود

داور اول:

عنوان و چکیده

۱-آیا در عنوان مطالعه، زمان، مکان و جامعه مورد مطالعه ذکر شده است؟ موضوعیت ندارد

توضیح عنوان پیشنهادی: سوء تغذیه در سالمندان ایران: یک مطالعه مرور سیستماتیک و متاآنالیز

با توجه به اینکه نوع مطالعه اختصاصا "مرور سیستماتیک **شیوع** سوء تغذیه" است. علیرغم احترام به نظر داور محترم محققین ترجیح می دهند عنوان به همان شکل باقی بماند زیرا به نظر می رسد حذف کلمه ی شیوه از عنوان باعث صدمه به گویایی و جامعیت عنوان شود.

۲-آیا عنوان انگلیسی با عنوان فارسی همخوانی دارد؟مشخص نشده

توضیح با توجه به عنوان فارسی اصلاح شود. با توجه به عنوان هم خوان شده است.

۳-آیا در چکیده پروپوزال، خلاصه بیان مساله و روش اجرا و کلمات کلیدی بطور صحیح و کافی ارائه شده است؟ بلی

توضیح پیشنهاد می شود در مورد چک لیست مورد استفاده برای استخراج داده ها و نرم افزار آماری مورد استفاده در متاآنالیز هم توضیح داده شود. همچنین در مورد اینکه در موارد عدم توافق بین دو نفر ارزیابی کننده چکار خواهند کرد توضیح دهند.

درمتن توضیحات بیشتری آورده شد.

بیان مساله، بررسی متون و اهداف

۴- آیا در بیان مساله، توضیحات کافی در مورد اهمیت موضوع، عوامل مرتبط با موضوع، دانش موجود در مورد موضوع و دلیل نیاز به انجام این مطالعه ارائه شده است؟ بلی

توضیح بهتر است در مورد ضرورت انجام این مطالعه و مزیت های نسبی آن نسبت به مرورهای قبلی توضیحات بیشتری داده شود.

توضیحات بیشتری در خصوص مزیت های نسبی این مطالعه نسبت به مطالعات قبلی اضافه شد.

۵- آیا در بررسی متون، منابع جدید مرتبط با موضوع بطور کافی استفاده شده و نتایج مثبت و منفی بطور کامل ارائه شده است؟ بلی

توضیح در برخی موراد که مطالعات قبلی توضیح داده شده اند به ابزارهای مورد استفاده در مطالعات اشاره نشده است.

موارد در متن اصلاح شد.

۶-آیا اهداف مطالعه با عنوان و بیان مساله مطابقت دارد؟ بلی

توضیح اهداف پیشنهادی: تعیین شیوع سوء تغذیه سالمندان ایران بر اساس کیفیت متدولوژیک مطالعات اولیه تعیین ناهمگونی متدولوژیک و علل بالقوه آن در سوء تغذیه سالمندان ایران

موارد در متن پروپوزال قید شده است.

روش اجرا

۸-آیا در روش اجرای مطالعه، نوع مطالعه بدرستی تعیین شده است؟ بلی

توضیح روش اجرای پروپوزال پیوست شده بسیار کاملتر از نسخه ای است که در پژوهشیار وارد شده است. بهتر است تکمیل شود.

موارد اضافه شد.

۹-آیا در جدول متغیرها، همه متغیرها مطابق با اهداف ذکر شده اند؟ خیر

توضیح جدول متغیرها در پژوهشیار وارد نشده است.

با توجه به اینکه مطالعه به صورت ثانویه انجام می شود موضوعیت ندارد

۱۲-آیا در مورد ابزار و روش جمع آوری داده ها (مطابق با جدول متغیرها) توضیحات کافی ارائه شده است؟ خیر

توضیح در مورد پایگاه های اطلاعاتی مورد استفاده توضیح داده نشده است. در مورد چک لیست استفاده شده برای ارزیابی کیفیت توضیح داده شود.

لیست پایگاهها در بخش روش کار آورده موجود است زیر خط دار شد. توضیحات بیشتر در خصوص ابزار هوی اضافه شد

۱۵-آیا در مورد محدودیتهای مطالعه توضیحات کافی ارائه شده است؟ خیر

توضیح عدم دسترسی به متن کامل یک مطالعه، عدم وجود نتایج مورد نیاز در مطالعات و عدم پاسخ ایمیل از طرف نویسندگان مقالات و نیز سوگیری های انتشار می تواند محدودیت های این مطالعه باشد.

تمامی موارد ذکر شده در فوق درمتن پروپوزال اشاره شده است

زمان بندی و هزینه های طرح

۱۷-آیا هزینه های پرسنلی بطور صحیح و مناسب تعیین شده است؟   خیر

توضیح   با توجه به اینکه طرح پایان نامه ای است و نیز توجه به این نکته که حداقل دو ارزیاب برای قسمتهای ارزیابی کیفیت و جستجو و نیز استخراج داده ها باید وجود داشته باشند، مجری محترم باید در جستجوی منابع و متاآنالیز و نیز در جستجوی منابع و ارزیابی کیفیت اصلاحاتی انجام دهند. همچنین هزینه های دانشجو و استاد کلا صفر شود و فقط برای کارشناس پروژه ای هزینه نوشته شود.

از آنجائیکه پایان نامه ی حاضر به طرح تحقیقاتی تبدیل شده است. هزینه ها منطبق بر قوانین معاونت تحقیقات برای حمایت از طرح های تحقیقاتی پایان نامه ای در نظر گرفته شده است.

۲۲-آیا مجموع هزینه های طرح بطور صحیح و مناسب محاسبه شده است؟   خیر

توضیح   قسمت هزینه های پرسنلی باید اصلاح شود.

موارد طبق قوانین معاونت تحقیقات در نظر گرفته شده است

داور دوم:

عنوان و چکیده

۳-آیا در چکیده پروپوزال، خلاصه بیان مساله و روش اجرا و کلمات کلیدی بطور صحیح و کافی ارائه شده است؟ بلی

توضیح   اهمیت موضوع و ضرورت (خلاصه بیان مسئله) بیان نشده است. روش اجرا بیان شده است نرم افزار و آزمون ها بیان نشده است.

موارد در متن پروپوزال اصلاح شد.

بیان مساله، بررسی متون و اهداف

۴- آیا در بیان مساله، توضیحات کافی در مورد اهمیت موضوع، عوامل مرتبط با موضوع، دانش موجود در مورد موضوع و دلیل نیاز به انجام این مطالعه ارائه شده است؟   بلی

توضیح   بیان مساله، توضیحات کافی در مورد اهمیت موضوع، عوامل مرتبط با موضوع، دانش موجود در مورد موضوع داده شده . نیاز با سازمانبندی پاراگراف ها دارد. مثلا بیان شود که مشکلات سالمندان ی چیست طبق آمار و سپس بیان شود که سو تغذیه مشکل رایج است. دلیل نیاز به انجام این مطالعه نیازمند توضیح بیشتر است.

موارد در متن پروپوزال اصلاح شد.

۵- آیا در بررسی متون، منابع جدید مرتبط با موضوع بطور کافی استفاده شده و نتایج مثبت و منفی بطور کامل ارائه شده است؟ خیر

توضیح بررسی متون بیان نشده است . ضرورت مطالعه با نقد بر متون بیان نشده است.

با توجه به فرمت جدید پروپوزال بیان مساله و بررسی متون به صورت ادغام شده و ضرورت اجرای مطالعه در انتهای بخش آورده شده است. با اینحال بنا به درخواست داور محترم در دو بخش جداگانه آورده شد.

روش اجرا

۹-آیا در جدول متغیرها، همه متغیرها مطابق با اهداف ذکر شده اند؟ خیر

توضیح جدول متغیرها بیان نشده است.

با توجه به نوع مطالعه موضوعیت ندارد

۱۰-آیا در جدول متغیرها، مشخصات متغیرها بطور صحیح ذکر شده اند؟ خیر

توضیح در جدول متغیرها، مشخصات متغیرها بطور صحیح ذکر نشده اند

با توجه به نوع مطالعه موضوعیت ندارد

۱۱-آیا در مورد جامعه مورد مطالعه، حجم نمونه و روش نمونه گیری توضیحات کافی ارائه شده است؟ بلی

توصیح در مورد جامعه مورد مطالعه، حجم نمونه و روش نمونه گیری توضیحات کافی ارائه شده است فقط محدوده زمانی مقالات مورد استفاده بیان شود.

در مطالعات مرور سیستماتیک شیوع معمولا انتهای بازه ی زمانی آورده می شود. در این مطالعه نیز محققین انتهای سپتامبر ۲۰۱۸ (شهریور ۱۳۹۷) را در متن پروپوزال ذکر کرده اند

۱۴-آیا در مورد ملاحظات اخلاقی توضیحات کافی ارائه شده است؟ خیر

توضیح توضیحات کافی نمی باشد.

با توجه به اینکه مطالعه برروی مطالعات اولیه انجام خواهد شد و نه آزمودنی های انسانی موارد اخلاقی که باید مد نظرقرار گیرد در متن آورده شده است.

۱۵-آیا در مورد محدودیتهای مطالعه توضیحات کافی ارائه شده است؟ بلی

توضیح در قسمت محدودیت و راهکار پیشنهادی اگر با نویسندگان مکاتبه شود و جواب گرفته نشود چه انجام می دهید نیز قید شود.

در بخش انتهایی پروپوزال اضافه شده است.

منابع

۲۳-آیا لیست منابع پروپوزال مطابق با راهنما بطور صحیح ارائه شده است؟ مشخص نشده

توضیح از منابع به روز استفاده شود. برخی منابع ۲۰۰۱ است .

با توجه به نوع مطالعه که مرور سیستماتیک شیوع است محققین ملزم هستند تمامی منابع که برخی حتی متعلق به سالهای ۱۹۸۰ هستند را نیز در مطالعه وارد کنند.

داور سوم:

عنوان و چکیده

۲-آیا عنوان انگلیسی با عنوان فارسی همخوانی دارد؟بلی

توضیح می توان خلاصه تر نوشت: Malnutrition Prevalence among Iranian Elderly People: A Systematic Review and Meta-Analysis

مورد در متن اصلاح شد.

روش اجرا

۱۱-آیا در مورد جامعه مورد مطالعه، حجم نمونه و روش نمونه گیری توضیحات کافی ارائه شده است؟ بلی

توصیح استفاده از گزارشات کشوری به شرطی که شرایط ورود به مطالعه را داشته باشد بهتر است ذکر شود سابقه وجود بیماریهای مزمن یا سرطان در افراد و یا وجود سرطان در حال حاضر در افراد مورد توجه قرار گیرد. تفاوت ابزارها بررسی سوء تغذیه چگونه است به نظر می رسد چند ابزار مطرح شده است

درمتن پروپوزال تحت عنوان سازمانهای ملی آورده شده است.

۱۲-آیا در مورد ابزار و روش جمع آوری داده ها (مطابق با جدول متغیرها) توضیحات کافی ارائه شده است؟ بلی

توضیح فیلد مورد استفاده در استراتژی جستجو بهتر است TIAB باشد بازه زمانی جستجو مشخص شود

با توجه به اینکه با تغییر فیلد تغییر چشمگیری در تعداد مقالات بازیابی شده ایجاد نشد. علیرغم احترام به نظر داور محترم ، ترجیح محققین به استفاده از فیلد " همه ی موارد" است علیرغم اینکه تعداد مقالات نهایی و حجم کار افراد مسئول غربالگری بسیار زیاد خواهد شد اما به این ترتیب موارد منفی کاذب به صفر نزدیکتر خواهد شد.

بجای بازه ی زمانی انتهای زمان جستجو در متن پروپوزال (در روش کار و چکیده روش کار) وارد شده است.

۱۳-آیا در مورد روش تجزیه و تحلیل داده ها، توضیحات کافی ارائه شده است؟ بلی

توضیح : هتروژنیتی بین مطالعات؟؟

مورد درمتن اصلاح شد.

۱۷-آیا هزینه های پرسنلی بطور صحیح و مناسب تعیین شده است؟   مشخص نشده

توضیح   به نظر می رسد هزینه پرسنلی برای استاد و دانشجو در پایان نامه پیش بینی نمی شود

با توجه به اینکه پایان نامه ی حاضر به طرح تحقیقاتی تبدیل شده ا ست موارد طبق قواعد معاونت تحقیقات و فن آوری دانشگاه در خصوص حمایت از طرح های تحقیقاتی در نظر گرفته شده است. البته هزینه برای دانشجو و اساتید مشاور و راهنما صفر شده است.

داور چهارم:

عنوان و چکیده

۱-آیا در عنوان مطالعه، زمان، مکان و جامعه مورد مطالعه ذکر شده است؟   خیر

توضیح   پیشنهاد می شود کلمه " یک مطالعه " از عنوان حذف شود.

با توجه به نوع مطالعه و ضرورت پیروی از الگوی استاندارد عنوان نویسی در این مطالعات، ضمن احترام به نظر داور محترم قابل حذف نمی باشد

۲-آیا عنوان انگلیسی با عنوان فارسی همخوانی دارد؟خیر

توضیح   درصورت قبول عنوان پیشنهادی جدید عنوان انگلیسی اصلاح شود.

۳-آیا در چکیده پروپوزال، خلاصه بیان مساله و روش اجرا و کلمات کلیدی بطور صحیح و کافی ارائه شده است؟ خیر

توضیح   ۱- پیشنهاد می شود محدوده زمانی معین تعیین شود (با توجه به منابع جستجوی در سالهای دور و روشهای تشخیصی مثلا ۳۰ سال قبل. ۲- در این بخش نیز به کلیه پایگاهها و منایع جستجو اشاره شود. ۳- به جای کلمه انگلیسی پیشنهاد می شود از کلمه لاتین یا کلمه بدون محدودیت زبان استفاده شود. ۴- در کلمات کلیدی پیشنهاد میشود اپیدمیولوژی اضافه شود. ۵- پیشنهاد می شود هر کدام از مراحل مطالعه توسط دو نفر و مستقل از هم و و در نهایت توافق نتایج توسط نفر سوم مورد بررسی قرارگیرد.

موارد ۱ تا ۳ درمتن اصلاح و زیر خط دار شد. مورد ۴ علیرغم احترام به داور محترم قابل تغییر نیست. مورد ۵ درمتن در ذیل هریک از مراحل اشاره شده است

.۶-آیا اهداف مطالعه با عنوان و بیان مساله مطابقت دارد؟   خیر

توضیح   با توجه به اینکه عوامل اجتماعی و اقتصادی در ایجاد مشکل سوء تغذیه نقش اساسی دارند پیشنهاد می شود به عنوان یک هدف فرعی مورد بررسی قرار گیرد

با توجه به نوع مطالعه، مرور مطالعات اولیه انجام شده محققین کنترلی بر روی متغیرهای اندازه گیری شده توسط محققین اولیه ندارند اما در صورتی که این متغیر توسط تعداد کافی (حداقل ۴-۵) مطالعه اولیه دارای کیفیت قابل قبول اندازه گیری شده باشد حتما در این مطالعه نیز مورد بررسی قرار خواهد گرفت.

۷-آیا سوالات و فرضیات با اهداف مطابقت دارد؟ خیر

توضیح در صورت پذیرش پیشنهاد در بند ۶ این بخش نیز اصلاح شود.

در فوق توضیح داده شد.

روش اجرا

۸-آیا در روش اجرای مطالعه، نوع مطالعه بدرستی تعیین شده است؟ خیر

توضیح به نوع مطالعه اشاره شده اما بنظر اینجانب روشن و شفاف نیست.

نوع مطالعه مرور نظام مند است که در متن به آن اشاره شده است

۹-آیا در جدول متغیرها، همه متغیرها مطابق با اهداف ذکر شده اند؟ خیر

توضیح در صورتیکه عوامل اجتماعی و اقتصادی در اهداف گنجانده شد در جول متغیرها آورده شود.

با توجه به نوع مطالعه موضوعیت ندارد.

۱۰-آیا در جدول متغیرها، مشخصات متغیرها بطور صحیح ذکر شده اند؟ خیر

توضیح در صورت پذیرش پیشنهاد بند ۹ این بخش نیز اصلا شود.

با توجه به نوع مطالعه موضوعیت ندارد.

۱۱-آیا در مورد جامعه مورد مطالعه، حجم نمونه و روش نمونه گیری توضیحات کافی ارائه شده است؟ خیر

توصیح در خصوص جامعه، حجم نمونه و روش نمونه گیری تا حدودی مطالب مناسب است به نظر می رسد شفافتر و واضحتر بیان شود بهتر است (بازنگری شود).

در بخش روش کار به طور کامل شرح داده شده است.

۱۲-آیا در مورد ابزار و روش جمع آوری داده ها (مطابق با جدول متغیرها) توضیحات کافی ارائه شده است؟ خیر

توضیح در مجموع مناسب است پیشنهاد می شود به کلیه پایگاههای اطلاعاتی اشاره شود

درمتن پروپوزال در بخش روش کار اشاره و زیر خط دار شده است

.

باتوجه به مطالعات مشابه در سالمندان بستری ، در خصوص مطالعاتی که شیوع را گزارش نکرده اند چرا از مقالات ونتایج این بخش استفاده نمی شود. ۲-در خصوص مطالعاتی که شیوع را گزارش نکرده اند چگونه داده های آن مورد استفاده قرار می گیرند؟

پیشنهاد داور محترم می تواندر مطالعه دیگری مورد بررسی قرار گیرد. در خصوص بخش دوم سوال، تنها از بخشی از یافته ها که شیوع را گزارش کرده باشند استفاده خواهد شد مثلا بخش اطلاعات پایه مطالعات کوهورت و مداخله ای. موارد در متن مورد اشاره قرار گرفته است.

با تشکر

استاد راهنما دکتر حمیرا خدام
